# Participatory logic modeling in a multi-site initiative to advance implementation science

**DOI:** 10.1186/s43058-023-00468-6

**Published:** 2023-08-29

**Authors:** Douglas V. Easterling, Rebekah R. Jacob, Ross C. Brownson, Debra Haire-Joshu, Daniel A. Gundersen, Heather Angier, Jennifer E. DeVoe, Sonja Likumahuwa-Ackman, Thuy Vu, Russell E. Glasgow, Robert Schnoll

**Affiliations:** 1 Department of Social Sciences and Health Policy, Wake Forest School of Medicine, Winston-Salem, NC 27157 USA; 2 Prevention Research Center, Brown School, Washington University in St. Louis, One Brookings Drive, Campus, Box 1196, St. Louis, MO 63130 USA; 3 Department of Surgery (Division of Public Health Sciences) and Alvin J. Siteman Cancer Center, Washington University School of Medicine, Washington University in St. Louis, St. Louis, MO 63130 USA; 4 Center for Diabetes Translation Research, Washington University in St. Louis, 1 Brookings Drive, Campus, Box 1196, St. Louis, MO 63117 USA; 5 Department of Medicine, Washington University School of Medicine, Washington University in St. Louis, St. Louis, MO 63130 USA; 6 Division of Population Sciences, Dana-Farber Cancer Institute, 450 Brookline Avenue, Boston, MA 02215 USA; 7 Department of Social and Behavioral Sciences, Harvard TH Chan School of Public Health, 677 Huntington Avenue, Boston, MA 02115 USA; 8 Vaccine and Infectious Disease Division, Fred Hutchinson Cancer Center, 1100 Fairview Ave N, Seattle, WA 98109 USA; 9 Department of Family Medicine, Oregon Health & Science University, 3181 SW Sam Jackson Park Road, Portland, OR 97239 USA; 10 Health Promotion Research Center, Department of Health Systems and Population Health, University of Washington, Seattle, WA 98195 USA; 11 Department of Family Medicine, University of Colorado, 1890 N Revere Ct, 3Rd Floor, Aurora, CO 80045 USA; 12 Department of Psychiatry and Abramson Cancer Center, University of Pennsylvania, 3535 Market Street, 4Th Floor, Philadelphia, PA 19104 USA

**Keywords:** Logic models, Participatory evaluation, Multi-site initiatives, Engaging grantees, Implementation science for cancer control, Health equity

## Abstract

**Background:**

Logic models map the short-term and long-term outcomes that are expected to occur with a program, and thus are an essential tool for evaluation. Funding agencies, especially in the United States (US), have encouraged the use of logic models among their grantees. They also use logic models to clarify expectations for their own funding initiatives. It is increasingly recognized that logic models should be developed through a participatory approach which allows input from those who carry out the program being evaluated. While there are many positive examples of participatory logic modeling, funders have generally not engaged grantees in developing the logic model associated with their own initiatives. This article describes an instance where a US funder of a multi-site initiative fully engaged the funded organizations in developing the initiative logic model. The focus of the case study is Implementation Science Centers in Cancer Control (ISC^3^), a multi-year initiative funded by the National Cancer Institute.

**Methods:**

The reflective case study was collectively constructed by representatives of the seven centers funded under ISC^3^. Members of the Cross-Center Evaluation (CCE) Work Group jointly articulated the process through which the logic model was developed and refined. Individual Work Group members contributed descriptions of how their respective centers reviewed and used the logic model. Cross-cutting themes and lessons emerged through CCE Work Group meetings and the writing process.

**Results:**

The initial logic model for ISC^3^ changed in significant ways as a result of the input of the funded groups. Authentic participation in the development of the logic model led to strong buy-in among the centers, as evidenced by their utilization. The centers shifted both their evaluation design and their programmatic strategy to better accommodate the expectations reflected in the initiative logic model.

**Conclusions:**

The ISC^3^ case study demonstrates how participatory logic modeling can be mutually beneficial to funders, grantees and evaluators of multi-site initiatives. Funded groups have important insights about what is feasible and what will be required to achieve the initiative’s stated objectives. They can also help identify the contextual factors that either inhibit or facilitate success, which can then be incorporated into both the logic model and the evaluation design. In addition, when grantees co-develop the logic model, they have a better understanding and appreciation of the funder’s expectations and thus are better positioned to meet those expectations.

Contributions to the literature The case study presented here provides a road map for engaging grantees in the development of an initiative-level logic model. While previous publications have reported on participatory logic modeling processes, these are generally for specific programs operating in a particular site. There are no published articles describing how grantees in a multi-site initiative have collaborated with the funding agency and initiative evaluator to develop a logic model that reflects all parties’ interests and expectations. From the funding agency perspective, the participatory process described here helped to ensure that all relevant outcomes are evaluated and enhance understanding, alignment, and buy-in across the grantees. For the grantees, this process enhanced collaboration and promoted common measures and outcomes. In turn, this effort has promoted a fuller and more active implementation of the initiative which addresses a longstanding implementation challenge facing funders of ambitious multi-site initiatives.

## Background

Logic models are one of the most important and widely used tools in the evaluation field. A logic model depicts the program designer’s expectations for what will occur and the mechanisms or pathways through which those outcomes will occur [1, 2]. Logic models can be applied to a broad range of “programs,” including direct service interventions, structured trainings, legislation, institutional policies, advocacy campaigns, community development initiatives, and research programs [2]. In addition, implementation scientists are increasingly relying on logic models to describe the expectations associated with the strategies used to implement programs [3–5]. Funders also use logic models to clarify their expectations when developing funding strategies and programmatic initiatives, as well as to communicate those expectations to grantees [6–8].

This article focuses specifically on logic modeling in the context of Implementation Science Centers in Cancer Control (ISC^3^), a multi-year initiative funded by the National Cancer Institute (NCI) that supports the development, testing and refinement of innovation approaches to implement evidence-based cancer control interventions. NCI is the United States (US) federal government’s principal agency for cancer research and training (https://www.cancer.gov/about-nci/overview). It is also the world’s largest funder of cancer research. Funding opportunities offered by NCI vary in terms of the types of research activities that can be supported and the specific requirements that awardees must meet. For ISC^3^, NCI used a P50 grant mechanism which is designed to support specialized research centers (https://grants.nih.gov/grants/funding/ac_search_results.htm?text_curr=p50&Search_Type=Activity). Seven university-based research groups were funded to develop and test implementation strategies that will improve cancer prevention and control. In addition to advancing implementation science within each of the funded institutions, NCI had the broader goal of expanding and strengthening the field of implementation science across the US.

### Logic model basics

Logic models organize the program designer’s expectations within a causal chain which typically includes the following domains: *inputs* (i.e., resources available to support a given program or study, such as human resources or finances), *activities* (i.e., actions taken to address the identified problem, concern, or need), *outputs* (i.e., products yielded from activities, including changes in knowledge and attitude, new or stronger relationships, coalition development, strategic plans, or new infrastructure for implementation), *outcomes* (i.e., tangible results spanning a temporal continuum and relating to the program’s goals, including behavior change, policy enactment, higher functioning organizations, or improved community capacity), and *impacts* (i.e., the ultimate pay-offs from the outcomes, such as changes in disease morbidity and mortality). Just as importantly, logic models use arrows to indicate the causal pathways through which outcomes and impacts are expected to occur.

The W.K. Kellogg Foundation popularized the use of logic models with two guidebooks published in 1998 [9] and 2003 [10], the first of which defined a logic model as “… a picture of how your program works – the theory and assumptions underlying the program….This model provides a roadmap of your program, highlighting how it is expected to work, what activities need to come before others, and how desired outcomes are achieved” ([9] p35). Such a roadmap is useful in guiding the choice of evaluation measures and methods as well as pointing out the specific hypotheses to test [1, 11].

The initial purpose motivating logic models was to ensure that program evaluations focus on the “right” outcomes and test the “right” underlying theories (i.e., those that the program designers had in mind) [10, 12, 13]. As evaluators began creating logic models with clients, it became apparent that this exercise brought value beyond guiding evaluation. Namely, the inquiry and conversation that goes along with creating a logic model often brings clarity and specificity to the program designers’ intent and assumptions [14].

### Participatory logic modeling

One of the most important advances in logic modeling was expanding the set of actors engaged in creating the logic model. Initially, logic models were generally drafted by evaluators who incorporated the expectations they elicited from program designers. This approach quickly gave way to one where program developers and funders created logic models as part of the design process (either with or without the support of an evaluator). With the advent of evaluation paradigms such as Participatory Evaluation, Collaborative Evaluation and Empowerment Evaluation in the 1990s, there was a widespread recognition that broader input is needed to produce valid logic models. According to the American Evaluation Association (AEA) [15], the US Centers for Disease Control and Prevention [16], and the Joint Commission on Standards for Educational Evaluation (JCSCEE) [17], one of the key principles of good evaluation is to “devote attention to the full range of individuals and groups invested in the program and affected by its evaluation.”

There are both practical and ethical reasons to engage the people and communities that are being served by a program or funding initiative when spelling out expected outcomes and causal pathways [18, 19]. They have a legitimate stake in determining what constitutes “success,” as well as real-world knowledge as to how and under what conditions the program’s outcomes are likely to occur [10]. For funder-designed initiatives, the organizations that receive funding have similar expertise as well as their own distinct interests which should be reflected in the logic model [20]. In addition, when program designers and funders co-develop the logic model with the people who will carry out the work, there will be greater alignment in expectations, allowing for fuller implementation [18].

The merits of participatory logic modeling have been recognized for at least two decades [19, 21, 22]. One excellent example is from Afifi et al. [18], who describe how a coalition of young people living in a Palestinian refugee camp in Lebanon designed a multi-level program to address the mental health needs of youth. The logic modeling process was an essential phase in both designing the program and determining how to evaluate it.

Although several examples of participatory logic modeling are described in the literature, they generally pertain to single *program* logic models rather than *multi-site initiative* logic models. In most funder initiatives, a small group of staff from the funding organization (e.g., the director of the initiative, an evaluation manager) develops an initial version of the logic model at the time the initiative is designed, and then this logic model is refined once an external evaluator is hired, usually through a collaborative process involving the funder and the evaluator. The initiative logic model is often shared with the groups that are funded under the initiative in order to provide a clearer sense of the funder’s intent and assumptions, but there generally are no opportunities for grantees to influence the logic model.

In some multi-site initiatives, the evaluation approach is described as “participatory” [23, 24], but the forms of participation are generally downstream from the logic modeling process, such as deciding which information to collect, providing data, administering surveys to program participants, and being an audience for findings from the evaluation. Rarely do funded groups have the opportunity to collaborate with the funder and the initiative evaluator to create or refine the initiative logic model.

### Logic modeling in the ISC^3^ initiative

ISC^3^ represents what we believe is the first documented case of a multi-site initiative where the funding agency actively engaged funded organizations in developing the initiative-level logic model. The ISC^3^ initiative, launched by NCI in 2019 and funded by the Beau Biden Cancer Moonshot^SM^ Initiative, funds seven centers for five years through a P50 mechanism. The initiative is designed to dramatically strengthen the national capacity to impact cancer prevention and control through implementation science [25]. ISC^3^ represents NCI’s largest investment to date focused on implementation science [26].

The seven ISC^3^ centers conduct research and build capacity for the use of implementation science across the cancer care continuum. Some centers were supported as “advanced centers” and others as “developing centers,” with varying award amounts, leadership structures, and foci. Building on prior NCI’s prior work in the area of IS, funded centers were expected to (1) establish IS “laboratories” to conduct collaborative research focused on testing implementation strategies to reduce cancer risk and improve cancer care [27]; (2) conduct rapid innovative projects to identify effective methods to improve the use of evidence-based programs in the context of cancer prevention and control; (3) develop resources, training, and mentorship to strengthen the national availability of implementation scientists and capacity for conducting implementation research; and (4) identify methods for cross-center collaboration to broaden the overall impact of the initiative.

Evaluation is strongly emphasized within ISC^3^. Each funded center has investigators who are specifically tasked with evaluating the center’s capacity-building activities and studies. The funding announcement required applicants to include a logic model that would demonstrate what they expected to accomplish with their grant—with regard both to activities and outcomes. In addition, NCI contracted with Westat (a consulting firm with expertise in program evaluation and project management) to carry out data collection and analysis to evaluate ISC^3^’s overall (initiative-wide) outcomes, including the production and dissemination of new scientific knowledge and tools, and the building of the field of IS, especially as it supports cancer prevention and control efforts.

A Cross-Center Evaluation (CCE) Work Group, comprised of representatives from the seven centers, NCI, and Westat, was convened early in the establishment of ISC^3^ to promote learning and coordination among the centers’ evaluators and to ensure that the initiative-wide evaluation was aligned with the center-specific evaluations. The CCE Work Group served as the forum for transforming the initial version of the logic model (original development described below) into a version that more fully reflected the aims and programming of the seven funded centers. Over time, this logic model evolved, especially to have an increased focus on health equity, and helped to frame individual center and the NCI’s expectations of key measures, outcomes, and impacts.

## Methods

This case study describes the process through which the ISC^3^ logic model was developed, refined, and used by the funded centers, NCI and Westat. The authors of the paper were members of the CCE Work Group where the logic model was developed and refined.

### Logic model development

The CCE Work Group has met approximately once per month since the outset of ISC^3^ to discuss evaluation-related topics, coordinate evaluation activities across sites, and plan collective projects. Rotating co-chairs representing two different ISC^3^ centers set the agenda and facilitate each meeting. NCI staff actively participate in these meetings, while also providing logistical support and taking notes. It is important to point out that NCI staff do not direct the conversation nor do they use the meetings as a venue for instructing participants on what their centers should do; instead, the grant agreement serves as the basis for all accountability expectations.

Discussion of the logic model was regularly included on the agenda during the first 3 years of the initiative and continues to be revisited periodically. During these discussions, representatives from all seven centers, as well as NCI staff, bring up thoughts, perspectives, or concerns regarding the adequacy of the logic model as a representation of the expectations associated with ISC^3^. Westat staff also participate in these meetings on a periodic basis. Meeting minutes are circulated to Work Group members and are also posted on a Confluence site accessible to all ISC^3^ investigators, NCI staff and Westat.

Review and revision of the logic model extended beyond the CCE Work Group’s own meetings. Work group members brought early versions of the logic model to their respective centers for discussion and to elicit recommendations. The initiative’s steering committee (comprised of the principal investigators from each center) and additional work groups also reviewed various versions of the logic model and provided recommendations for revising. The CCE Work Group was responsible for reconciling the various input and creating subsequent versions of the logic model.

### Case study method

Members of the CCE Work Group conducted a reflective case study of the logic-model development process. A reflective case study is one where researchers document and analyze their own experience [28]. The case study was constructed according to the following steps:

1. The CCE Work Group collectively constructed an outline of the topics to be covered in the case study, including the process through which the logic model was developed and refined, the various ways in which the logic model was used, and the benefits and challenges associated with using a participatory process.
2. A subgroup of the CCE Work Group wrote an initial draft of how the logic model was developed and refined. That draft was distributed among other Work Group members (including representatives from NCI and Westat) who offered additional information and comments. The description included here incorporated that input as well as points raised during discussions in Work Group meetings.
3. Members of the CCE Work Group were asked to contribute information regarding their respective centers’ discussion and use of the logic model. That information was organized according to (a) promoting understanding and alignment, (b) guiding evaluation, and (c) guiding strategy.
4. Cross-cutting themes, implications, and lessons were generated through discussion in monthly meetings of the CCE Work Group, captured in meeting notes, and refined further in the collective writing of this manuscript. Notably, these discussions included representatives of NCI as well as the funded centers.

## Results

### Logic model development

**Fig. 1:**
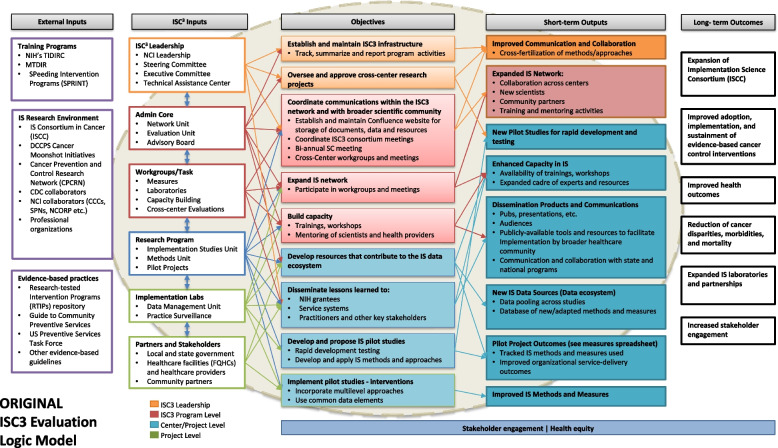
Original version of the ISC^3^ logic model

The initial draft of the initiative logic model (Fig. 1) was jointly created by NCI and Westat based on NCI’s expectations for ISC^3^ (as specified in the request for applications). Westat also incorporated the activities, outcomes, and measures that were included in the center-specific logic models and evaluation plans that were included in the funded proposals. The initiative logic model aggregated the center-specific activities and outcomes into a more global picture, while also representing initiative-wide inputs, activities and outcomes.

The initial version of the logic model was presented for review to the CCE Work Group in May of 2020. Both NCI and Westat encouraged feedback and suggestions. Work Group members offered a variety of ideas for making the logic model more comprehensive and easier to comprehend. After that meeting, one of the Work Group members (DVE) developed a mock-up of how the logic model might be structured to emphasize the primary causal pathways. This version was discussed at the next CCE Work Group meeting, stimulating further discussion and suggestions. In particular, the CCE Work Group recommended a variety of additions and revisions. Some of these were specific, including adding a box for the expected outcomes from the pilot projects, adding rapid cycle testing and implementation as a feature of the funded pilot projects, and embedding pilot projects within the implementation laboratories. A broader recommendation was to bring health equity more explicitly into both the activities and outcomes boxes of the model. Following this meeting, Westat and NCI conferred on how to incorporate the Work Group’s input into the official logic model for ISC^3^. They developed the next version, which maintained the basic form used in Fig. 1, while also including a large number of features that emerged in the two meetings and the mock-up version. That revised version was presented, discussed, and endorsed at the subsequent CCE Work Group meeting.

**Fig. 2:**
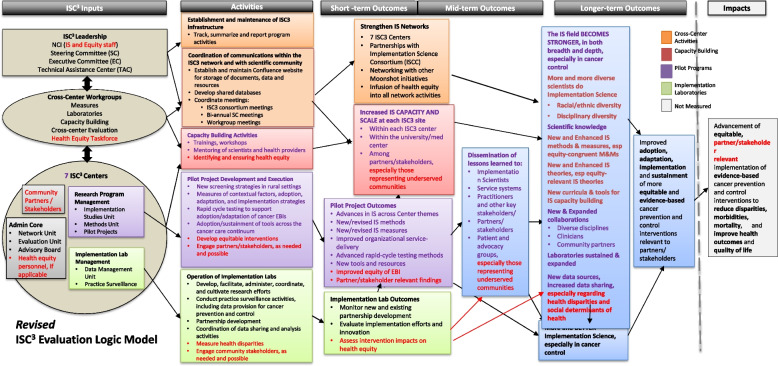
Revised version of the ISC^3^ logic model, highlighting health equity components

At the same time that they endorsed the revised logic model, the Work Group also determined that this should be a “living document” to be updated as the centers’ work continued to unfold. In fact, the activities and expectations associated with ISC^3^ have evolved in important ways during the implementation process. The current version of the initiative logic model is shown in Fig. 2. The CCE Work Group has continued to use a participatory process to accommodate these refinements, in each case involving actors from throughout the initiative. These include the overall initiative steering committee, other ISC^3^ Work Groups (i.e., for the Implementation Science Laboratories; Health Equity), and the investigators at each center. At each step, those reviewing the logic model have been invited to recommend additions or changes to the logic model.

### Incorporating health equity

One of the most substantial changes in the logic model was the increased focus on health equity which occurred during the first year of ISC^3^. As shown in Fig. 2, explicit references to health equity were added throughout the logic model, including the activities that the centers are expected to carry out (e.g., at least some pilot projects should emphasize equitable interventions), the expected short-term outcomes (e.g., increases in capacity should extend to partners who represent underserved communities), and the expected longer-term outcomes (e.g., increased diversity in the field of implementation scientists, new IS theories and methods grounded in equity principles).

These changes in the logic model occurred at the same time that NCI and investigators within the funded centers were having in-depth conversations around the role of health equity within IS. For example, health equity had been a major focus within NCI’s Consortium for Cancer Implementation Science (CCIS), a national network convened in 2019 to identify activities and products that would promote progress on key IS topics [29]. One the action groups formed under CCIS is “Health Equity and Context” and has membership that overlaps with ISC^3^. In addition, a number of individuals associated with ISC^3^ were writing articles pointing out that more and better equity-oriented tools, methods, conceptual frameworks, and trainings are needed if the IS field is to achieve its potential for improving health outcomes and reducing disparities [29–31].

Health equity has been included as an element of ISC^3^ from the outset. For example, the funding announcement issued in November 2018 required applicants to describe how their trainings would “reduce disparities in cancer prevention and control of traditionally underserved populations” (https://grants.nih.gov/grants/guide/rfa-files/rfa-ca-19-005.html). Health equity increased in prominence as the centers began carrying out their work and collaborating [25]. It was formally recognized as a priority theme when NCI and the ISC^3^ steering committee collaboratively decided to establish the ISC^3^ Health Equity Task Force in January 2021 as a mechanism to explicitly incorporate health equity into the design and implementation of ISC^3^.

This decision came shortly after the first annual grantee meeting in September 2020 where health equity had been a major topic of conversation. Those conversations were energized by the race-based hate crimes that occurred earlier in the year, especially the murder of George Floyd on May 25. However, it is important to note that many of the funded centers had an explicit focus on health equity research which predated their participation in ISC^3^.

The Health Equity Task Force determined that the logic model could provide a useful point of reference for assessing where health equity was already reflected within ISC^3^’s expectations and priorities and where health equity could be incorporated more explicitly. One key factor in this decision was the overlapping membership between the Task Force and the CCE Work Group. The Task Force also engaged the CCE Work Group in conversations to determine how the design of ISC^3^ should change so that the initiative would promote progress on health equity outcomes.

The Task Force developed a set of themes as to how health equity should be advanced within ISC^3^, each of which were incorporated into an updated version of the logic model. With guidance from the Task Force, the CCE Work Group devoted several monthly meetings to name specific health equity-oriented elements to be added to the inputs, activities, outcomes, and impacts.

These additions were verified and refined through conversations at the seven centers. Each center was tasked with asking their own center members for logic model feedback that the CCE Work Group then reviewed, discussed, and ultimately incorporated into the logic model. Based on this feedback, several refinements were made regarding where to include health equity and how to be more explicit with the outputs we are assessing. We continued to engage and seek input from the Task Force throughout this process. The work group decided that regular input from ISC^3^ leaders, work groups, and centers would ensure that updates to the model were in line with initiative activities. One additional idea that came up was discussion around how to explicitly include the engagement of community-based partners in the centers’ work, for example, with the implementation laboratories. These equity-related augmentations to the logic model are highlighted in red in the logic model shown in Fig. 2.

### Promoting understanding and alignment

The process of reviewing and augmenting the logic model yielded a more accurate logic model and also greater clarity among those involved in ISC^3^ around what was expected of funded centers in terms of activities and outcomes. This occurred within each of the seven funded centers as the logic model was reviewed and critiqued in team meetings. Table 1 presents examples of the expectations that were clarified and aligned within individual centers.

**Table 1:** Examples of how the initiative logic model was used by funded centers

Use	Center	Example
1) Promoted Understanding and alignment	IDAPT Center (Wake Forest University and University of Massachusetts)	Clarified expectations around the types of partners who should participate in the Lab, as well the mentoring of junior faculty
	Washington University at St. Louis	Engaged in a formal process to refine their center’s logic model to ensure alignment with the overall initiative model
	Penn ISC^3^ (University of Pennsylvania)	Required that each project was co-led by a junior and senior research to align with the initiative logic model’s priority of training and mentorship
	OPTICC (University of Washington)	Facilitated alignment in vision among the 3 investigators (from 2 different institutions) leading the center—by identifying how core activities were distinct or overlapped, which facilitated communication and aligned evaluation activities
	Harvard Implementation Science Center for Cancer Control Equity (ISCCCE)	Community partners were engaged in defining how equity is most relevant and salient in their practices, and then subsequently in carrying out shared work
	Oregon Health Sciences University (OHSU)	Logic model was used to communicate the center’s goals and emphasis on equity to co-investigators and partners in the Lab
	Colorado ISC^3^ (University of Colorado)	Recognized that more emphasis was needed on capacity building, rapid adaptations and dissemination within the Lab
2) Guided Evaluation	Initiative-wide	Key methods for the initiative evaluation (Annual Grantee Survey, Collaboration Survey) were revised to align with the logic model
	Harvard Implementation Science Center for Cancer Control Equity (ISCCCE)	Data capture protocol for monitoring the center’s activities and scientific products was revised to better align with the outputs listed in the logic model and requested in the Annual Grantee Survey
	Washington University at St. Louis	Identified additional aspects of scientists’ social networks to assess when conducting center-specific analyses of Collaboration Survey
	Penn ISC^3^ (University of Pennsylvania)	Included measures of health equity in progress reporting formats used by all research studies
	IDAPT Center (Wake Forest University and University of Massachusetts)	Guided the development of the interview guide for interviews with junior investigators supported by IDAPT
	Oregon Health Sciences University (OHSU)	The initiative model was used to identify short-, mid-, and longer-term outputs from the Center’s work
3) Guided Strategy	Colorado ISC^3^ (University of Colorado)	Developed interactive and web-based tools to help guide scientists, practitioners, and community implementation teams to plan for success, dissemination and sustainability, and also to inform iterative adaptations
	Washington University at St. Louis	Revised the Center’s request for applications to focus on health equity and cross-center capacity building. Added community members as reviewers of pilot applications
	Colorado ISC^3^ (University of Colorado) and Washington University at St. Louis	Updated the dissemination-implementation.org website to include constructs and examples related to health equity
	Penn ISC^3^ (University of Pennsylvania)	Developed request for applications that prioritized studies that addressed health equity and selected studies that focus on cancer-relevant health equity
	Oregon Health Sciences University (OHSU)	Expanded research and dissemination partnerships to include a Lab focused on Latino health disparities and equity in primary care. Added regular monitoring of disparities in cancer screening and prevention. Secured a diversity supplement to host a graduate fellow in the center
	OPTICC (University of Washington)	Reinforced the importance of incorporating Lab partners’ priorities (especially around health equity) into the selection of pilot awards and evaluating the effectiveness of pilot projects (e.g., special focus on reducing disparities in colorectal cancer screening in the FQHC project
	Harvard Implementation Science Center for Cancer Control Equity (ISCCCE)	Increased emphasis on cross-center partnerships in pilot grants and manuscripts. Pilot program added equity as a factor in the review of applications

One of the key insights that emerged involved the specificity of the activities, outputs, and outcomes. Some of NCI’s expectations were quite specific (e.g., an expanded and more densely connected network of IS researchers, training more researchers and clinicians in IS methods, new IS measures and tools). In contrast, some elements of ISC^3^, particularly the Implementation Lab, had more generically defined outcomes in the logic model, with the expectation that each Center would develop its own strategy to achieve outcomes directly relevant to the center and its clinical partners.

### Guiding evaluation

The logic model is the primary point of reference in determining evaluation methods and measures for both the initiative-level evaluation and the local evaluations conducted by each center.

#### Initiative-wide evaluation

Westat relied on the logic model to develop the Annual Grantee Survey, which is the primary method used in the initiative-wide evaluation of ISC^3^. This survey asks representatives from each center to report on the programmatic activities, including progress on the studies funded; securing extramural funding for new investigator-initiated research; publications and presentations; laboratory expansion; training, mentoring, and other forms of capacity building; and the development of new methods, theories and tools; and the outcomes of those activities. The logic model pointed to the important activities and outcomes, ensuring consistency across the centers in reporting content. The Annual Grantee Survey was revised in year 2 of the initiative to include new questions reflecting the health equity elements added to the logic model. For example, in the section focused on evaluating the outcomes from center studies, the following question was added: Do studies include health-equity focused components, targets, or outcomes? The following question was also added: To what extent are ISC^3^ outputs being disseminated to patient and advocacy groups–-especially those representing underserved communities?

A second key method used in the initiative-wide evaluation is the Collaboration Survey, which supports a social network analysis of investigators engaged in IS work within and across the centers [32]. Questions in the survey are aligned with relevant outcomes in the logic model (e.g., strengthen IS networks). As health equity became a more central focus of ISC^3^, new analyses were conducted to assess the position of under-represented scientists in the network.

#### Center-specific evaluations

As a complement to the initiative-level evaluation carried out by Westat, each center conducts evaluations of its own programming. The center-specific logic models provided the initial guidance for these “local” evaluations. As the ISC^3^ logic model took shape, it allowed leadership at each center to refine their evaluation plans to be more fully aligned with the initiative’s expectations and priorities. As a result, centers made changes to their interview guides, reporting forms for pilot awards and data-capture processes, while also identifying new research questions and topics to address when analyzing these data. Specific examples are shown in Table 1.

### Guiding strategy

As leaders of each center reviewed the logic model, they sometimes recognized that their existing ISC^3^ strategy was not “complete” in terms of meeting expectations for either activities or outcomes. As shown in Table 1, this led to a number of enhancements or revisions in the activities that the centers carried out. Many of the changes were made in response to the increased emphasis on health equity within the logic model.

The pilot award program was frequently the focus of these changes. A number of centers added equity as an explicit review factor and/or added community members as reviewers. Capacity-building strategies were also enhanced so as to reach more diverse audiences and to include health equity as a key topic when discussing implementation science methods, theories, and principles.

## Discussion

ISC^3^ is distinct from other multi-site initiatives in that the funded centers have been equal partners with the funder and the evaluator in developing and defining the initiative logic model. Representatives from each of the funded centers have worked collectively and collaboratively with representatives from NCI and Westat to develop and revise the initiative’s logic model. In the first 2 years of the initiative, the logic model changed in significant ways due to this collaborative process, with representatives from all funded centers having influence over its design. Moreover, the process pointed to opportunities to expand and strengthen the design of ISC^3^, again in line with the shared interests of NCI and the seven centers. Benefits and lessons from the case study are summarized in Table 2.

**Table 2:** Benefits and lessons from the ISC^3^ case study

Topic	Lesson
Improving the Logic Model and the Evaluation Process	Directly engaging funded groups produced an initiative logic model with more complete specification of activities, outcomes and pathways
	The resultant logic model better reflected the expectations and understanding of the funded groups, without any diminishment in the representation of the funder’s expectations and understanding of the initiative
	The enhancements to the logic model resulting from the participatory process pointed to concepts that were not fully captured in the original set of evaluation measures
Collateral Benefits	Actively reviewing and editing the logic model allowed investigators at the funded centers to more fully understand and align with the funder’s expectations
	Discussions about the logic model led to more alignment around strategy and objectives within each center
Requirements	The participatory process required each funded center to have at least 1 representative willing to focus on this task over an extended period of time. Their responsibilities included not only actively participating in the co-development process but also serving as a liaison to others within their center who have a stake and/or relevant knowledge
	Because of the iterative nature of the co-development process, it took approximately 6 months to move from the original logic model to agreement on the first revision
	For initiatives that evolve in their goals and/or design, co-development of the logic model should continue
	Having representatives from the funded centers who were skilled in research and evaluation was a distinct advantage in co-developing the ISC^3^ logic model
	The funder’s openness and ethic of collaboration were critical in ensuring that the logic model actually evolved in line with grantees’ input
	The participatory process required more time and effort from the evaluation firm than was initially budgeted for in the development of the logic model

### Benefits

The participatory process allowed the logic model to reflect the funder’s expectations and theory of change. as well as the perspective and interests of the groups responsible for carrying out the work that the funder envisioned. Input from the ISC^3^ centers clarified and refined the expected outcomes and the pathways through which those outcomes will occur. The centers had the authority to question the funding agency’s assumptions and to operationalize those assumptions and even to propose additional lines of equity-oriented work and outcomes that were supported under ISC^3^. Authentic participation in the development of the logic model led to strong buy-in among the centers. The centers shifted their evaluation design and their programmatic strategy to better accommodate the expectations reflected in the logic model.

This case study demonstrates that engaging funded groups can lead to more specific and realistic logic models, which has important benefits for both evaluation and strategy of large scale and multi-site implementation science initiatives. Those doing the work (i.e., closest to the ground) have important insights about what is feasible and what will be required to achieve the initiative’s stated objectives. They can also help identify the contextual factors that either inhibit or facilitate success, which can then be incorporated into the logic model and the evaluation design. As the logic model becomes more accurate and grounded, the funder may ways to enhance the design of the initiative. To the extent that such expansions are included, the initiative will be more potent and more likely to achieve its goals and objectives. In addition, when grantees co-develop the logic model, they have a better understanding and appreciation of the funder’s expectations, and thus are better positioned to meet those expectations.

### Lessons

Engaging grantees in the development of an initiative logic model is admittedly challenging because of the chicken-and-egg dilemma. How can grantees participate in developing the logic model if they have not yet been selected? The ISC^3^ case study resolves this dilemma by demonstrating that no matter how thoughtful the funder is prior to the launching of an initiative, the logic model will inherently be a first approximation. The logic model can be improved by revisiting it with grantees once they have been selected and begun implementing the initiative.

Another challenge with participatory logic modeling is the requirements imposed on grantees. In many initiatives, the funded organizations do not have representatives with evaluation expertise. ISC^3^ was unique in this regard: the RFA required each center to include an evaluator as part of its leadership team. Other NIH initiatives with similar requirements [33, 34] could replicate the participatory logic modeling process used in ISC^3^. Engaging grantee representatives in logic modeling is admittedly more difficult in initiatives where the funded organizations are small nonprofits or grassroots groups.

Even in cases where the funded groups have evaluation expertise, participatory logic modeling can be challenging because of the time required to review, discuss, revise and reach agreement, especially for complex initiatives such as ISC^3^. Time is required not only from grantees, but also the funder and the evaluator. There are opportunity costs for each; time spent clarifying and refining the logic model takes away from other evaluation-related tasks, as well as other work needed to achieve the initiative’s desired outcomes. The funder may also need to include extra funds for the external evaluator to accommodate a participatory process.

One other consideration worth mentioning is that the participatory approach profiled here required a genuine commitment from the funder to participate as an equal partner in revising the logic model. NCI staff actively engaged in the process, offering well-reasoned advice on what to include and how to frame specific concepts. At the same time, they explicitly stated that this was a collective process and that they would not dictate the final product. In fact, the concept for this paper and its content emerged independent of the funders influence. This orientation on the part of NCI staff was crucial in mitigating the power imbalance that often arises when a funder enters into collaborative work with its grantees. Not all funders are this open to grantee input.

## Conclusions

The ISC^3^ case demonstrates that by engaging funded groups in the logic modeling task, funders can actually better achieve their own goals. The groups carrying out the work specified in the initiative have a clear sense of which goals are feasible, what it will take to reach those goals, and how the funder can best contribute [35]. Grantees’ knowledge and perspective produces a more accurate logic model, more informed evaluation methods and measures, and even a more effective and efficient funding strategy. We hope that the ISC^3^ case study provides a positive example of how participatory logic modeling can be mutually beneficial to funders, grantees, and evaluators of multi-site initiatives. While we believe that many of our lessons apply in various global settings, it is likely that adaptations to our process will be needed to match local context.

## Data Availability

Materials related to the case study, including additional versions of the logic model, are available from the corresponding author upon reasonable request.

## Abbreviations

ISC^3^: Implementation Science Centers for Cancer Control
NCI: National Cancer Institute
CCE: Cross-Center Evaluation (Work Group)
